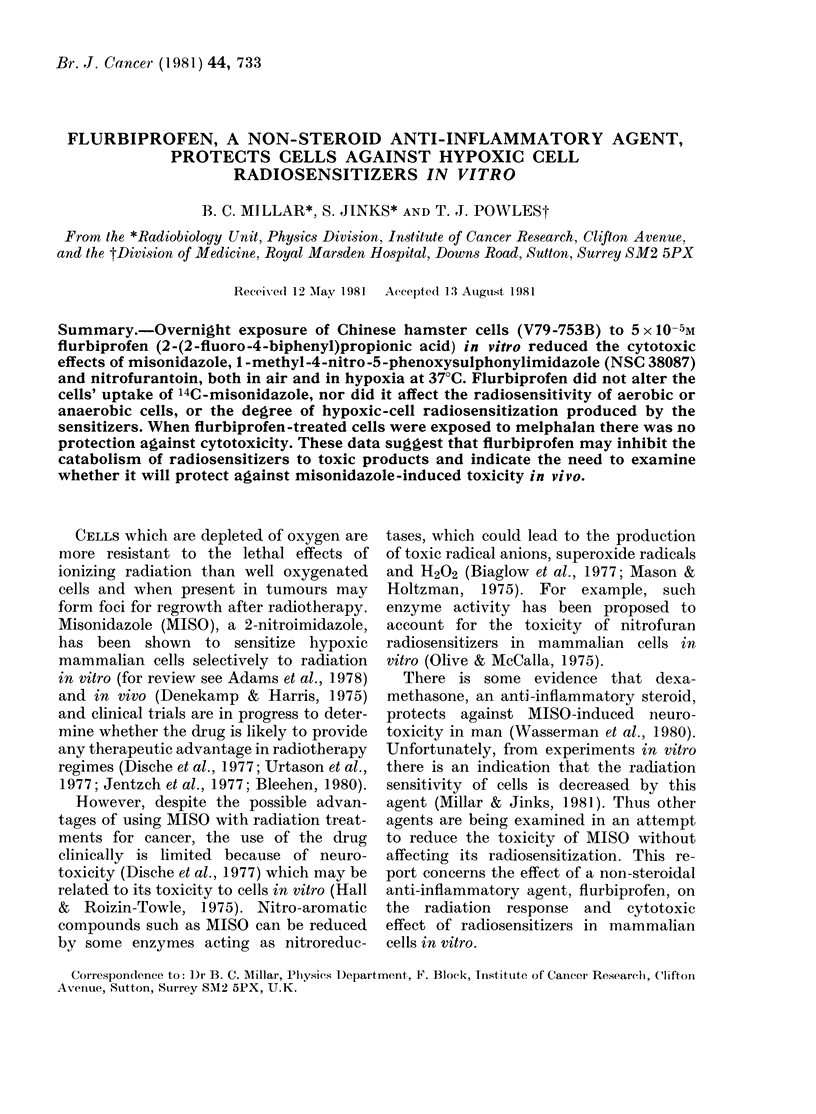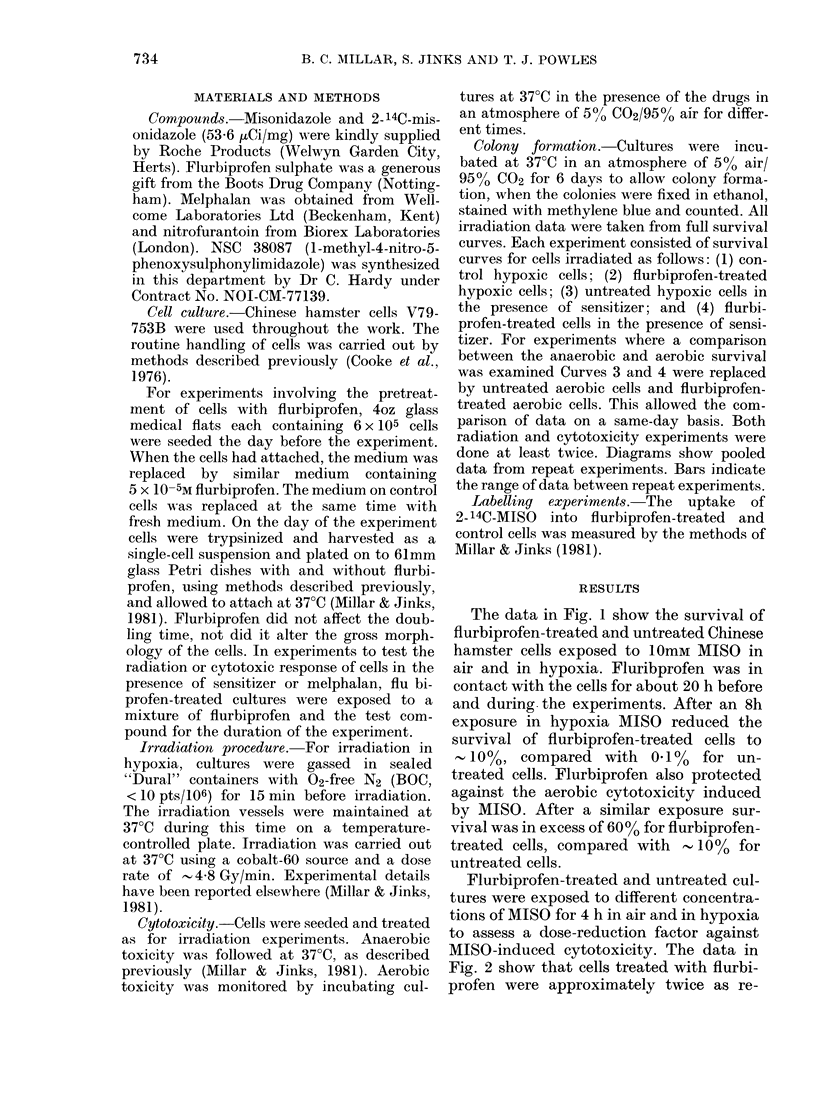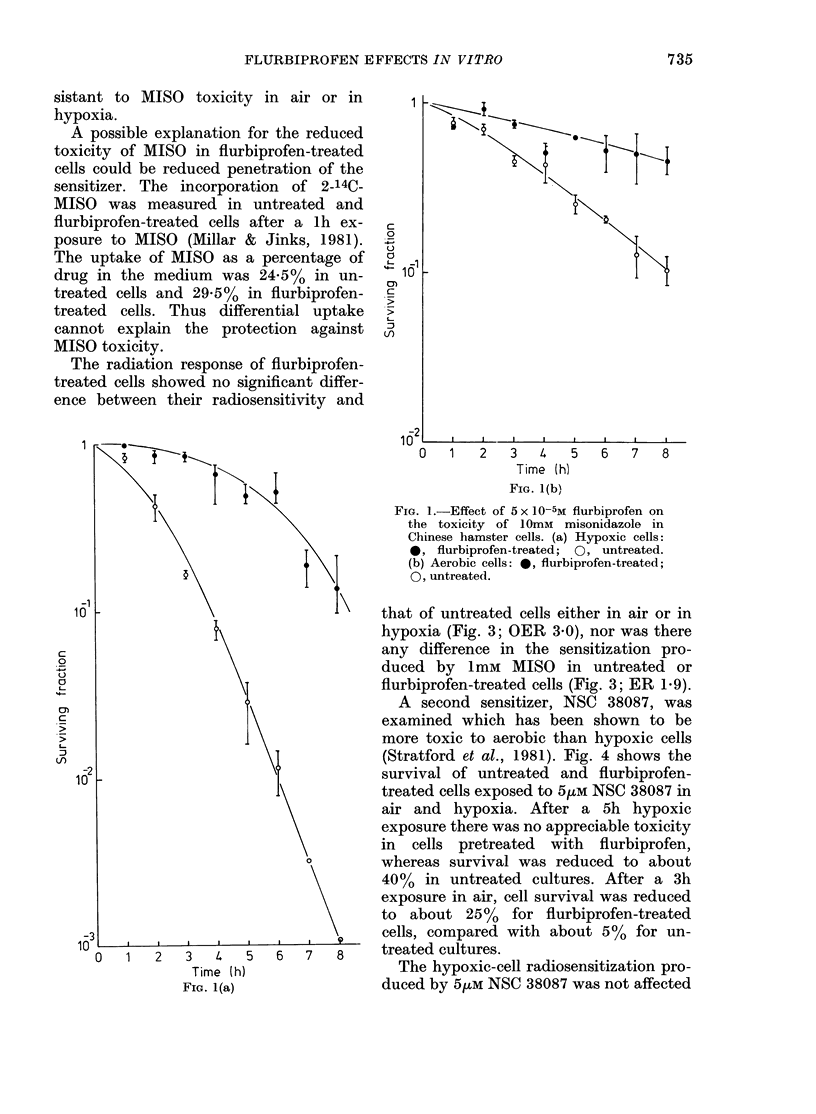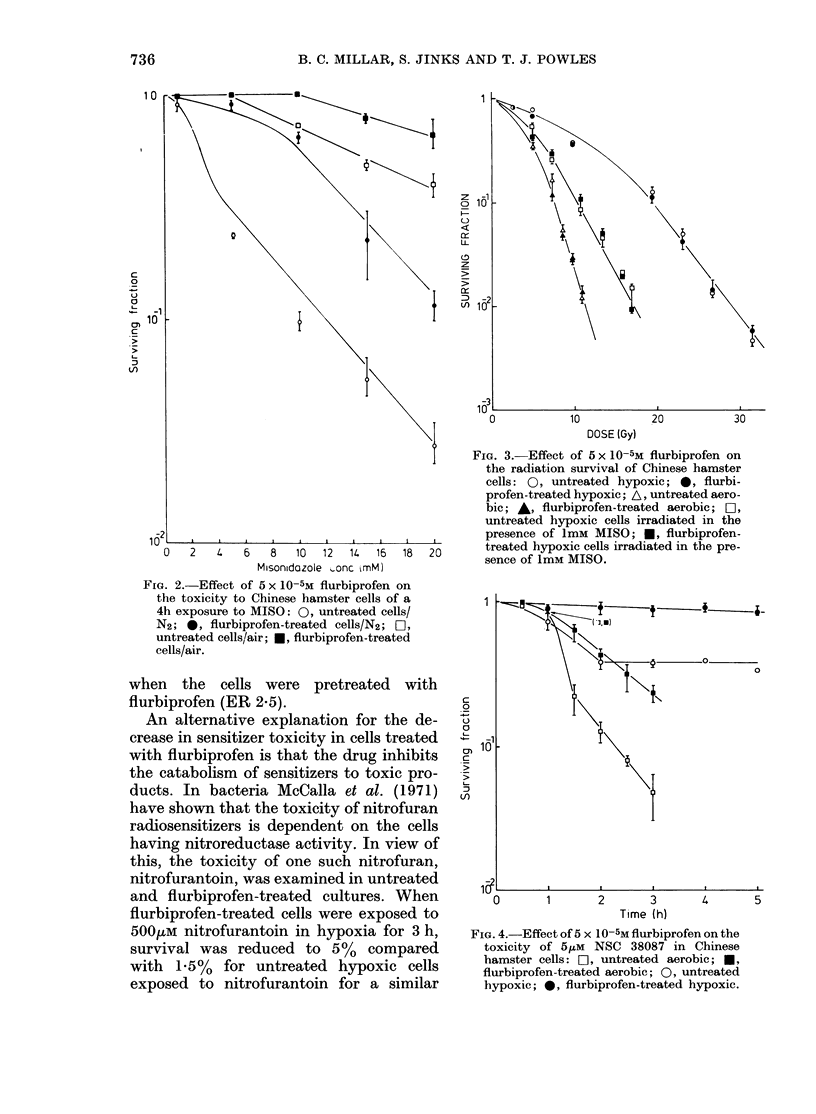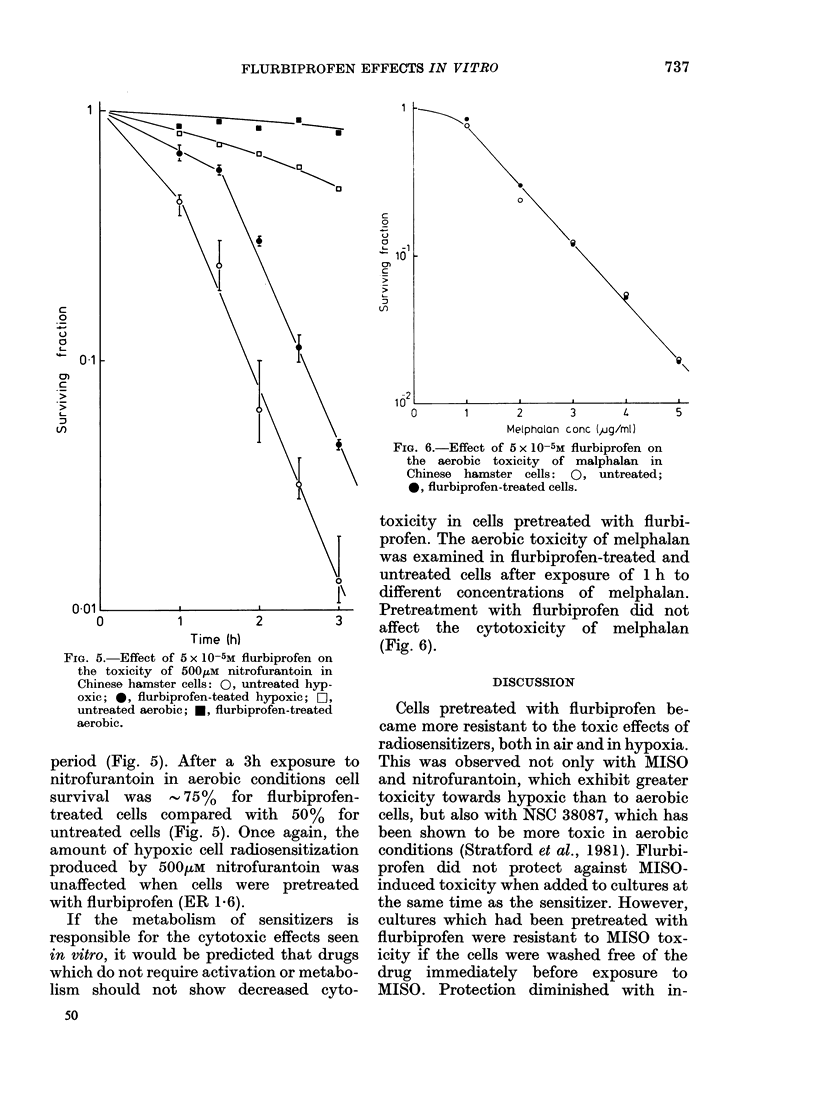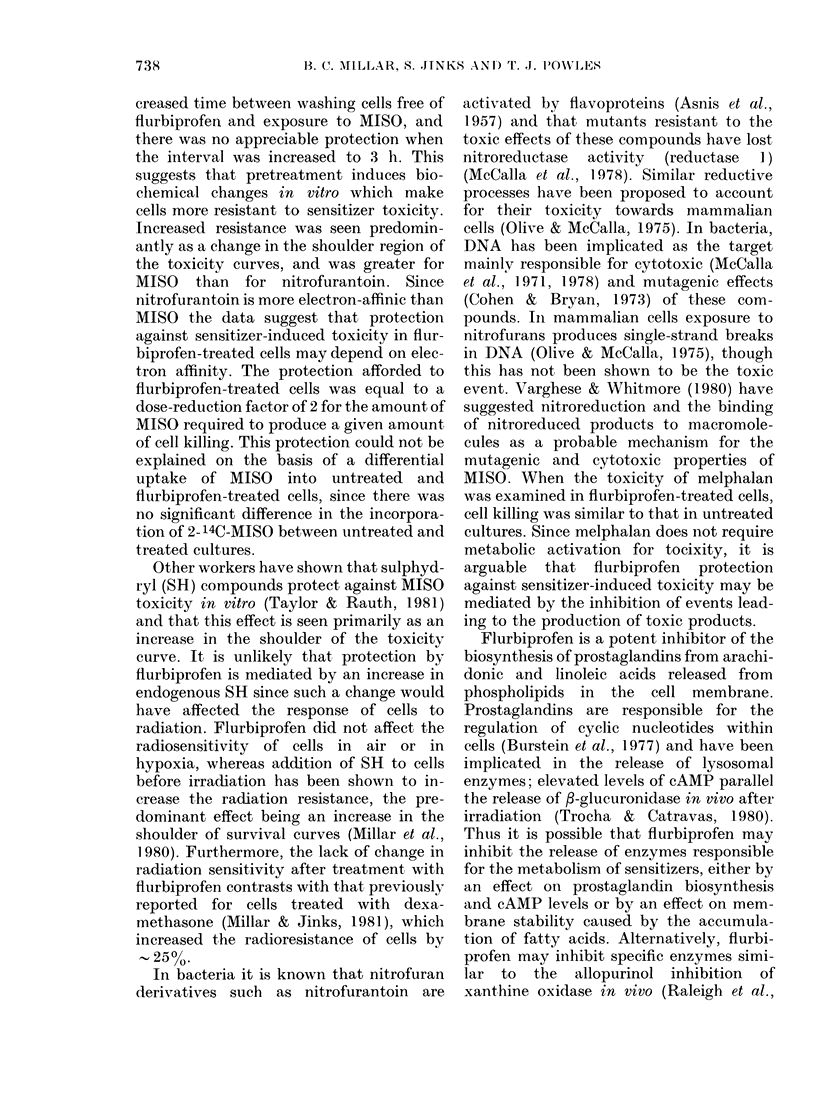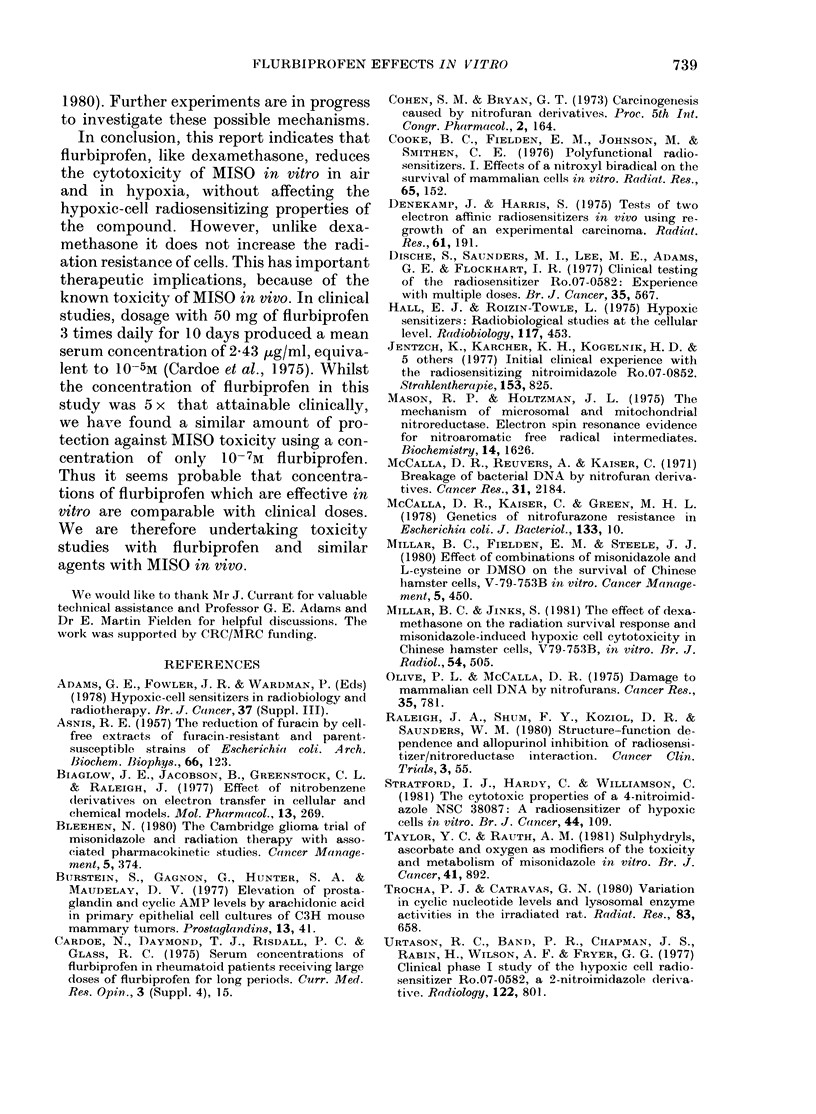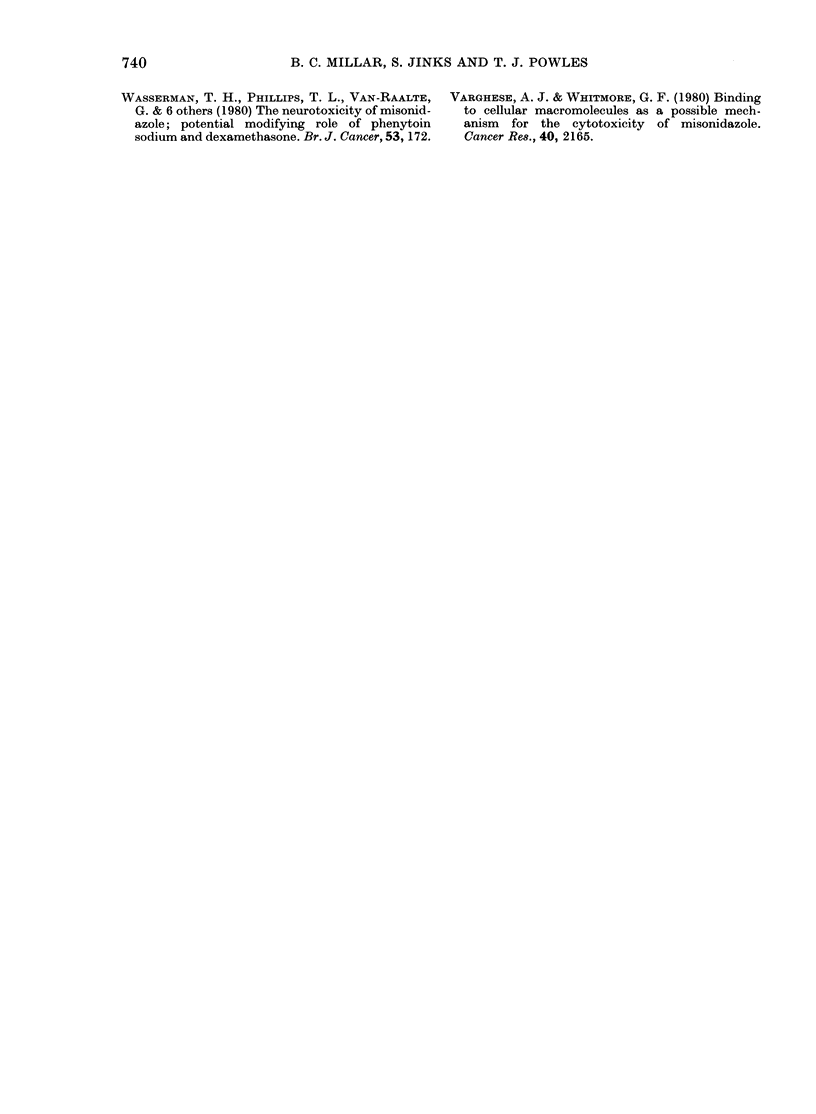# Flurbiprofen, a non-steroid anti-inflammatory agent, protects cells against hypoxic cell radiosensitizers in vitro.

**DOI:** 10.1038/bjc.1981.260

**Published:** 1981-11

**Authors:** B. C. Millar, S. Jinks, T. J. Powles

## Abstract

Overnight exposure of Chinese hamster cells (V79-753B) to 5 x 10(-5) M flurbiprofen (2-(2-fluoro-4-biphenyl)propionic acid) in vitro reduced the cytotoxic effects of misonidazole, 1-methyl-4-nitro-5-phenoxysulphonylimidazole (NSC 38087) and nitrofurantoin, both in air and in hypoxia at 37 degrees C. Flurbiprofen did not alter the cells' uptake of 14C-misonidazole, nor did it affect the radiosensitivity of aerobic or anaerobic cells, or the degree of hypoxic-cell radiosensitization produced by the sensitizers. When flurbiprofen-treated cells were exposed to melphalan there was no protection against cytotoxicity. These data suggest that flurbiprofen may inhibit the catabolism of radiosensitizers to toxic products and indicate the need to examine whether it will protect against misonidazole-induced toxicity in vivo.


*Br. J. Cancer* (1981) **44**, 733

### FLURBIPROFEN, A NON-STEROID ANTI-INFLAMMATORY AGENT, PROTECTS CELLS AGAINST HYPOXIC CELL RADIOSENSITIZERS *IN VITRO*

B. C. MILLAR*, S. JINKS* AND T. J. POWLES†

*From the *Radiobiology Unit, Physics Division, Institute of Cancer Research, Clifton Avenue, and the †Division of Medicine, Royal Marsden Hospital, Downs Road, Sutton, Surrey SM2 5PX*

Received 12 May 1981 Accepted 13 August 1981

**Summary.—Overnight exposure of Chinese hamster cells (V79-753B) to $5 \times 10^{-5}$M flurbiprofen (2-(2-fluoro-4-biphenyl)propionic acid) *in vitro* reduced the cytotoxic effects of misonidazole, 1-methyl-4-nitro-5-phenoxysulphonylimidazole (NSC 38087) and nitrofurantoin, both in air and in hypoxia at 37°C. Flurbiprofen did not alter the cells' uptake of $^{14}$C-misonidazole, nor did it affect the radiosensitivity of aerobic or anaerobic cells, or the degree of hypoxic-cell radiosensitization produced by the sensitizers. When flurbiprofen-treated cells were exposed to melphalan there was no protection against cytotoxicity. These data suggest that flurbiprofen may inhibit the catabolism of radiosensitizers to toxic products and indicate the need to examine whether it will protect against misonidazole-induced toxicity *in vivo*.**

CELLS which are depleted of oxygen are more resistant to the lethal effects of ionizing radiation than well oxygenated cells and when present in tumours may form foci for regrowth after radiotherapy. Misonidazole (MISO), a 2-nitroimidazole, has been shown to sensitize hypoxic mammalian cells selectively to radiation *in vitro* (for review see Adams *et al.*, 1978) and *in vivo* (Denekamp & Harris, 1975) and clinical trials are in progress to determine whether the drug is likely to provide any therapeutic advantage in radiotherapy regimes (Dische *et al.*, 1977; Urtason *et al.*, 1977; Jentzch *et al.*, 1977; Bleehen, 1980).

However, despite the possible advantages of using MISO with radiation treatments for cancer, the use of the drug clinically is limited because of neurotoxicity (Dische *et al.*, 1977) which may be related to its toxicity to cells *in vitro* (Hall & Roizin-Towle, 1975). Nitro-aromatic compounds such as MISO can be reduced by some enzymes acting as nitroreductases, which could lead to the production of toxic radical anions, superoxide radicals and $H_2O_2$ (Biaglow *et al.*, 1977; Mason & Holtzman, 1975). For example, such enzyme activity has been proposed to account for the toxicity of nitrofuran radiosensitizers in mammalian cells *in vitro* (Olive & McCalla, 1975).

There is some evidence that dexamethasone, an anti-inflammatory steroid, protects against MISO-induced neurotoxicity in man (Wasserman *et al.*, 1980). Unfortunately, from experiments *in vitro* there is an indication that the radiation sensitivity of cells is decreased by this agent (Millar & Jinks, 1981). Thus other agents are being examined in an attempt to reduce the toxicity of MISO without affecting its radiosensitization. This report concerns the effect of a non-steroidal anti-inflammatory agent, flurbiprofen, on the radiation response and cytotoxic effect of radiosensitizers in mammalian cells *in vitro*.

Correspondence to: Dr B. C. Millar, Physics Department, F. Block, Institute of Cancer Research, Clifton Avenue, Sutton, Surrey SM2 5PX, U.K.

### MATERIALS AND METHODS

*Compounds.*—Misonidazole and 2-$^{14}$C-misonidazole (53·6 $\mu$Ci/mg) were kindly supplied by Roche Products (Welwyn Garden City, Herts). Flurbiprofen sulphate was a generous gift from the Boots Drug Company (Nottingham). Melphalan was obtained from Wellcome Laboratories Ltd (Beckenham, Kent) and nitrofurantoin from Biorex Laboratories (London). NSC 38087 (1-methyl-4-nitro-5-phenoxysulphonylimidazole) was synthesized in this department by Dr C. Hardy under Contract No. NOI-CM-77139.

*Cell culture.*—Chinese hamster cells V79-753B were used throughout the work. The routine handling of cells was carried out by methods described previously (Cooke *et al.*, 1976).

For experiments involving the pretreatment of cells with flurbiprofen, 4oz glass medical flats each containing $6 \times 10^5$ cells were seeded the day before the experiment. When the cells had attached, the medium was replaced by similar medium containing $5 \times 10^{-5}$M flurbiprofen. The medium on control cells was replaced at the same time with fresh medium. On the day of the experiment cells were trypsinized and harvested as a single-cell suspension and plated on to 61mm glass Petri dishes with and without flurbiprofen, using methods described previously, and allowed to attach at 37°C (Millar & Jinks, 1981). Flurbiprofen did not affect the doubling time, not did it alter the gross morphology of the cells. In experiments to test the radiation or cytotoxic response of cells in the presence of sensitizer or melphalan, flu biprofen-treated cultures were exposed to a mixture of flurbiprofen and the test compound for the duration of the experiment.

*Irradiation procedure.*—For irradiation in hypoxia, cultures were gassed in sealed "Dural" containers with $O_2$-free $N_2$ (BOC, $< 10$ pts/$10^6$) for 15 min before irradiation. The irradiation vessels were maintained at 37°C during this time on a temperature-controlled plate. Irradiation was carried out at 37°C using a cobalt-60 source and a dose rate of $\sim$4·8 Gy/min. Experimental details have been reported elsewhere (Millar & Jinks, 1981).

*Cytotoxicity.*—Cells were seeded and treated as for irradiation experiments. Anaerobic toxicity was followed at 37°C, as described previously (Millar & Jinks, 1981). Aerobic toxicity was monitored by incubating cultures at 37°C in the presence of the drugs in an atmosphere of 5% $CO_2$/95% air for different times.

*Colony formation.*—Cultures were incubated at 37°C in an atmosphere of 5% air/95% $CO_2$ for 6 days to allow colony formation, when the colonies were fixed in ethanol, stained with methylene blue and counted. All irradiation data were taken from full survival curves. Each experiment consisted of survival curves for cells irradiated as follows: (1) control hypoxic cells; (2) flurbiprofen-treated hypoxic cells; (3) untreated hypoxic cells in the presence of sensitizer; and (4) flurbiprofen-treated cells in the presence of sensitizer. For experiments where a comparison between the anaerobic and aerobic survival was examined Curves 3 and 4 were replaced by untreated aerobic cells and flurbiprofen-treated aerobic cells. This allowed the comparison of data on a same-day basis. Both radiation and cytotoxicity experiments were done at least twice. Diagrams show pooled data from repeat experiments. Bars indicate the range of data between repeat experiments.

*Labelling experiments.*—The uptake of 2-$^{14}$C-MISO into flurbiprofen-treated and control cells was measured by the methods of Millar & Jinks (1981).

### RESULTS

The data in Fig. 1 show the survival of flurbiprofen-treated and untreated Chinese hamster cells exposed to 10mM MISO in air and in hypoxia. Fluribprofen was in contact with the cells for about 20 h before and during the experiments. After an 8h exposure in hypoxia MISO reduced the survival of flurbiprofen-treated cells to $\sim$10%, compared with 0·1% for untreated cells. Flurbiprofen also protected against the aerobic cytotoxicity induced by MISO. After a similar exposure survival was in excess of 60% for flurbiprofen-treated cells, compared with $\sim$10% for untreated cells.

Flurbiprofen-treated and untreated cultures were exposed to different concentrations of MISO for 4 h in air and in hypoxia to assess a dose-reduction factor against MISO-induced cytotoxicity. The data in Fig. 2 show that cells treated with flurbiprofen were approximately twice as resistant to MISO toxicity in air or in hypoxia.

A possible explanation for the reduced toxicity of MISO in flurbiprofen-treated cells could be reduced penetration of the sensitizer. The incorporation of 2-$^{14}$C-MISO was measured in untreated and flurbiprofen-treated cells after a 1h exposure to MISO (Millar & Jinks, 1981). The uptake of MISO as a percentage of drug in the medium was 24·5% in untreated cells and 29·5% in flurbiprofen-treated cells. Thus differential uptake cannot explain the protection against MISO toxicity.

The radiation response of flurbiprofen-treated cells showed no significant difference between their radiosensitivity and

FIG. 1(a)

FIG. 1(b)

FIG. 1.—Effect of $5 \times 10^{-5}$M flurbiprofen on the toxicity of 10mM misonidazole in Chinese hamster cells. (a) Hypoxic cells: ●, flurbiprofen-treated; ○, untreated. (b) Aerobic cells: ●, flurbiprofen-treated; ○, untreated.

that of untreated cells either in air or in hypoxia (Fig. 3; OER 3·0), nor was there any difference in the sensitization produced by 1mM MISO in untreated or flurbiprofen-treated cells (Fig. 3; ER 1·9).

A second sensitizer, NSC 38087, was examined which has been shown to be more toxic to aerobic than hypoxic cells (Stratford *et al.*, 1981). Fig. 4 shows the survival of untreated and flurbiprofen-treated cells exposed to 5$\mu$M NSC 38087 in air and hypoxia. After a 5h hypoxic exposure there was no appreciable toxicity in cells pretreated with flurbiprofen, whereas survival was reduced to about 40% in untreated cultures. After a 3h exposure in air, cell survival was reduced to about 25% for flurbiprofen-treated cells, compared with about 5% for untreated cultures.

The hypoxic-cell radiosensitization produced by 5$\mu$M NSC 38087 was not affected

FIG. 2.—Effect of $5 \times 10^{-5}$M flurbiprofen on the toxicity to Chinese hamster cells of a 4h exposure to MISO: ○, untreated cells/$N_2$; ●, flurbiprofen-treated cells/$N_2$; □, untreated cells/air; ■, flurbiprofen-treated cells/air.

FIG. 3.—Effect of $5 \times 10^{-5}$M flurbiprofen on the radiation survival of Chinese hamster cells: ○, untreated hypoxic; ●, flurbiprofen-treated hypoxic; △, untreated aerobic; ▲, flurbiprofen-treated aerobic; □, untreated hypoxic cells irradiated in the presence of 1mM MISO; ■, flurbiprofen-treated hypoxic cells irradiated in the presence of 1mM MISO.

when the cells were pretreated with flurbiprofen (ER 2·5).

An alternative explanation for the decrease in sensitizer toxicity in cells treated with flurbiprofen is that the drug inhibits the catabolism of sensitizers to toxic products. In bacteria McCalla *et al.* (1971) have shown that the toxicity of nitrofuran radiosensitizers is dependent on the cells having nitroreductase activity. In view of this, the toxicity of one such nitrofuran, nitrofurantoin, was examined in untreated and flurbiprofen-treated cultures. When flurbiprofen-treated cells were exposed to 500$\mu$M nitrofurantoin in hypoxia for 3 h, survival was reduced to 5% compared with 1·5% for untreated hypoxic cells exposed to nitrofurantoin for a similar

FIG. 4.—Effect of $5 \times 10^{-5}$M flurbiprofen on the toxicity of 5$\mu$M NSC 38087 in Chinese hamster cells: □, untreated aerobic; ■, flurbiprofen-treated aerobic; ○, untreated hypoxic; ●, flurbiprofen-treated hypoxic.

FIG. 5.—Effect of $5 \times 10^{-5}$M flurbiprofen on the toxicity of 500$\mu$M nitrofurantoin in Chinese hamster cells: ○, untreated hypoxic; ●, flurbiprofen-teated hypoxic; □, untreated aerobic; ■, flurbiprofen-treated aerobic.

FIG. 6.—Effect of $5 \times 10^{-5}$M flurbiprofen on the aerobic toxicity of malphalan in Chinese hamster cells: ○, untreated; ●, flurbiprofen-treated cells.

period (Fig. 5). After a 3h exposure to nitrofurantoin in aerobic conditions cell survival was ~75% for flurbiprofen-treated cells compared with 50% for untreated cells (Fig. 5). Once again, the amount of hypoxic cell radiosensitization produced by 500$\mu$M nitrofurantoin was unaffected when cells were pretreated with flurbiprofen (ER 1·6).

If the metabolism of sensitizers is responsible for the cytotoxic effects seen *in vitro*, it would be predicted that drugs which do not require activation or metabolism should not show decreased cytotoxicity in cells pretreated with flurbiprofen. The aerobic toxicity of melphalan was examined in flurbiprofen-treated and untreated cells after exposure of 1 h to different concentrations of melphalan. Pretreatment with flurbiprofen did not affect the cytotoxicity of melphalan (Fig. 6).

### DISCUSSION

Cells pretreated with flurbiprofen became more resistant to the toxic effects of radiosensitizers, both in air and in hypoxia. This was observed not only with MISO and nitrofurantoin, which exhibit greater toxicity towards hypoxic than to aerobic cells, but also with NSC 38087, which has been shown to be more toxic in aerobic conditions (Stratford *et al.*, 1981). Flurbiprofen did not protect against MISO-induced toxicity when added to cultures at the same time as the sensitizer. However, cultures which had been pretreated with flurbiprofen were resistant to MISO toxicity if the cells were washed free of the drug immediately before exposure to MISO. Protection diminished with increased time between washing cells free of flurbiprofen and exposure to MISO, and there was no appreciable protection when the interval was increased to 3 h. This suggests that pretreatment induces biochemical changes *in vitro* which make cells more resistant to sensitizer toxicity. Increased resistance was seen predominantly as a change in the shoulder region of the toxicity curves, and was greater for MISO than for nitrofurantoin. Since nitrofurantoin is more electron-affinic than MISO the data suggest that protection against sensitizer-induced toxicity in flurbiprofen-treated cells may depend on electron affinity. The protection afforded to flurbiprofen-treated cells was equal to a dose-reduction factor of 2 for the amount of MISO required to produce a given amount of cell killing. This protection could not be explained on the basis of a differential uptake of MISO into untreated and flurbiprofen-treated cells, since there was no significant difference in the incorporation of 2-$^{14}$C-MISO between untreated and treated cultures.

Other workers have shown that sulphydryl (SH) compounds protect against MISO toxicity *in vitro* (Taylor & Rauth, 1981) and that this effect is seen primarily as an increase in the shoulder of the toxicity curve. It is unlikely that protection by flurbiprofen is mediated by an increase in endogenous SH since such a change would have affected the response of cells to radiation. Flurbiprofen did not affect the radiosensitivity of cells in air or in hypoxia, whereas addition of SH to cells before irradiation has been shown to increase the radiation resistance, the predominant effect being an increase in the shoulder of survival curves (Millar *et al.*, 1980). Furthermore, the lack of change in radiation sensitivity after treatment with flurbiprofen contrasts with that previously reported for cells treated with dexamethasone (Millar & Jinks, 1981), which increased the radioresistance of cells by $\sim 25\%$.

In bacteria it is known that nitrofuran derivatives such as nitrofurantoin are activated by flavoproteins (Asnis *et al.*, 1957) and that mutants resistant to the toxic effects of these compounds have lost nitroreductase activity (reductase 1) (McCalla *et al.*, 1978). Similar reductive processes have been proposed to account for their toxicity towards mammalian cells (Olive & McCalla, 1975). In bacteria, DNA has been implicated as the target mainly responsible for cytotoxic (McCalla *et al.*, 1971, 1978) and mutagenic effects (Cohen & Bryan, 1973) of these compounds. In mammalian cells exposure to nitrofurans produces single-strand breaks in DNA (Olive & McCalla, 1975), though this has not been shown to be the toxic event. Varghese & Whitmore (1980) have suggested nitroreduction and the binding of nitroreduced products to macromolecules as a probable mechanism for the mutagenic and cytotoxic properties of MISO. When the toxicity of melphalan was examined in flurbiprofen-treated cells, cell killing was similar to that in untreated cultures. Since melphalan does not require metabolic activation for tocixity, it is arguable that flurbiprofen protection against sensitizer-induced toxicity may be mediated by the inhibition of events leading to the production of toxic products.

Flurbiprofen is a potent inhibitor of the biosynthesis of prostaglandins from arachidonic and linoleic acids released from phospholipids in the cell membrane. Prostaglandins are responsible for the regulation of cyclic nucleotides within cells (Burstein *et al.*, 1977) and have been implicated in the release of lysosomal enzymes; elevated levels of cAMP parallel the release of $\beta$-glucuronidase *in vivo* after irradiation (Trocha & Catravas, 1980). Thus it is possible that flurbiprofen may inhibit the release of enzymes responsible for the metabolism of sensitizers, either by an effect on prostaglandin biosynthesis and cAMP levels or by an effect on membrane stability caused by the accumulation of fatty acids. Alternatively, flurbiprofen may inhibit specific enzymes similar to the allopurinol inhibition of xanthine oxidase *in vivo* (Raleigh *et al.*,

1980). Further experiments are in progress to investigate these possible mechanisms.

In conclusion, this report indicates that flurbiprofen, like dexamethasone, reduces the cytotoxicity of MISO *in vitro* in air and in hypoxia, without affecting the hypoxic-cell radiosensitizing properties of the compound. However, unlike dexamethasone it does not increase the radiation resistance of cells. This has important therapeutic implications, because of the known toxicity of MISO *in vivo*. In clinical studies, dosage with 50 mg of flurbiprofen 3 times daily for 10 days produced a mean serum concentration of 2·43 $\mu$g/ml, equivalent to $10^{-5}$M (Cardoe *et al.*, 1975). Whilst the concentration of flurbiprofen in this study was 5× that attainable clinically, we have found a similar amount of protection against MISO toxicity using a concentration of only $10^{-7}$M flurbiprofen. Thus it seems probable that concentrations of flurbiprofen which are effective *in vitro* are comparable with clinical doses. We are therefore undertaking toxicity studies with flurbiprofen and similar agents with MISO *in vivo*.

We would like to thank Mr J. Currant for valuable technical assistance and Professor G. E. Adams and Dr E. Martin Fielden for helpful discussions. The work was supported by CRC/MRC funding.

### REFERENCES

ADAMS, G. E., FOWLER, J. R. & WARDMAN, P. (Eds) (1978) Hypoxic-cell sensitizers in radiobiology and radiotherapy. *Br. J. Cancer*, **37** (Suppl. III).

ASNIS, R. E. (1957) The reduction of furacin by cell-free extracts of furacin-resistant and parent-susceptible strains of *Escherichia coli*. *Arch. Biochem. Biophys.*, **66**, 123.

BIAGLOW, J. E., JACOBSON, B., GREENSTOCK, C. L. & RALEIGH, J. (1977) Effect of nitrobenzene derivatives on electron transfer in cellular and chemical models. *Mol. Pharmacol.*, **13**, 269.

BLEEHEN, N. (1980) The Cambridge glioma trial of misonidazole and radiation therapy with associated pharmacokinetic studies. *Cancer Management*, **5**, 374.

BURSTEIN, S., GAGNON, G., HUNTER, S. A. & MAUDELAY, D. V. (1977) Elevation of prostaglandin and cyclic AMP levels by arachidonic acid in primary epithelial cell cultures of C3H mouse mammary tumors. *Prostaglandins*, **13**, 41.

CARDOE, N., DAYMOND, T. J., RISDALL, P. C. & GLASS, R. C. (1975) Serum concentrations of flurbiprofen in rheumatoid patients receiving large doses of flurbiprofen for long periods. *Curr. Med. Res. Opin.*, **3** (Suppl. 4), 15.

COHEN, S. M. & BRYAN, G. T. (1973) Carcinogenesis caused by nitrofuran derivatives. *Proc. 5th Int. Congr. Pharmacol.*, **2**, 164.

COOKE, B. C., FIELDEN, E. M., JOHNSON, M. & SMITHEN, C. E. (1976) Polyfunctional radiosensitizers. I. Effects of a nitroxyl biradical on the survival of mammalian cells *in vitro*. *Radiat. Res.*, **65**, 152.

DENEKAMP, J. & HARRIS, S. (1975) Tests of two electron affinic radiosensitizers *in vivo* using regrowth of an experimental carcinoma. *Radiat. Res.*, **61**, 191.

DISCHE, S., SAUNDERS, M. I., LEE, M. E., ADAMS, G. E. & FLOCKHART, I. R. (1977) Clinical testing of the radiosensitizer Ro.07-0582: Experience with multiple doses. *Br. J. Cancer*, **35**, 567.

HALL, E. J. & ROIZIN-TOWLE, L. (1975) Hypoxic sensitizers: Radiobiological studies at the cellular level. *Radiobiology*, **117**, 453.

JENTZCH, K., KARCHER, K. H., KOGELNIK, H. D. & 5 others (1977) Initial clinical experience with the radiosensitizing nitroimidazole Ro.07-0852. *Strahlentherapie*, **153**, 825.

MASON, R. P. & HOLTZMAN, J. L. (1975) The mechanism of microsomal and mitochondrial nitroreductase. Electron spin resonance evidence for nitroaromatic free radical intermediates. *Biochemistry*, **14**, 1626.

MCCALLA, D. R., REUVERS, A. & KAISER, C. (1971) Breakage of bacterial DNA by nitrofuran derivatives. *Cancer Res.*, **31**, 2184.

MCCALLA, D. R., KAISER, C. & GREEN, M. H. L. (1978) Genetics of nitrofurazone resistance in *Escherichia coli*. *J. Bacteriol.*, **133**, 10.

MILLAR, B. C., FIELDEN, E. M. & STEELE, J. J. (1980) Effect of combinations of misonidazole and L-cysteine or DMSO on the survival of Chinese hamster cells, V-79-753B *in vitro*. *Cancer Management*, **5**, 450.

MILLAR, B. C. & JINKS, S. (1981) The effect of dexamethasone on the radiation survival response and misonidazole-induced hypoxic cell cytotoxicity in Chinese hamster cells, V79-753B, *in vitro*. *Br. J. Radiol.*, **54**, 505.

OLIVE, P. L. & MCCALLA, D. R. (1975) Damage to mammalian cell DNA by nitrofurans. *Cancer Res.*, **35**, 781.

RALEIGH, J. A., SHUM, F. Y., KOZIOL, D. R. & SAUNDERS, W. M. (1980) Structure–function dependence and allopurinol inhibition of radiosensitizer/nitroreductase interaction. *Cancer Clin. Trials*, **3**, 55.

STRATFORD, I. J., HARDY, C. & WILLIAMSON, C. (1981) The cytotoxic properties of a 4-nitroimidazole NSC 38087: A radiosensitizer of hypoxic cells *in vitro*. *Br. J. Cancer*, **44**, 109.

TAYLOR, Y. C. & RAUTH, A. M. (1981) Sulphydryls, ascorbate and oxygen as modifiers of the toxicity and metabolism of misonidazole *in vitro*. *Br. J. Cancer*, **41**, 892.

TROCHA, P. J. & CATRAVAS, G. N. (1980) Variation in cyclic nucleotide levels and lysosomal enzyme activities in the irradiated rat. *Radiat. Res.*, **83**, 658.

URTASON, R. C., BAND, P. R., CHAPMAN, J. S., RABIN, H., WILSON, A. F. & FRYER, G. G. (1977) Clinical phase I study of the hypoxic cell radiosensitizer Ro.07-0582, a 2-nitroimidazole derivative. *Radiology*, **122**, 801.

WASSERMAN, T. H., PHILLIPS, T. L., VAN-RAALTE, G. & 6 others (1980) The neurotoxicity of misonidazole; potential modifying role of phenytoin sodium and dexamethasone. *Br. J. Cancer*, **53**, 172.

VARGHESE, A. J. & WHITMORE, G. F. (1980) Binding to cellular macromolecules as a possible mechanism for the cytotoxicity of misonidazole. *Cancer Res.*, **40**, 2165.